# Progress Toward Measles Elimination — South-East Asia Region, 2003–2013

**Published:** 2015-06-12

**Authors:** Arun Thapa, Sudhir Khanal, Umid Sharapov, Virginia Swezy, Tika Sedai, Alya Dabbagh, Paul Rota, James L. Goodson, Jeffrey McFarland

**Affiliations:** 1 Expanded Programme on Immunization, World Health Organization South-East Asia Regional Office, Delhi, India; 2 Global Immunization Division, Center for Global Health, CDC; 3 Department of Immunization, Vaccines, and Biologicals, World Health Organization, Geneva, Switzerland; 4 Division of Viral Diseases, National Center for Immunization and Respiratory Diseases, CDC

In 2013, the 66th session of the Regional Committee of the World Health Organization (WHO) South-East Asia Region* adopted the goal of measles elimination and rubella and congenital rubella syndrome control† by 2020 after rigorous prior consultations (1–3). The recommended strategies include 1) achieving and maintaining ≥95% coverage with 2 doses of measles- and rubella-containing vaccine in every district through routine or supplementary immunization activities (SIAs)§; 2) developing and sustaining a sensitive and timely case-based measles surveillance system that meets recommended performance indicators¶; 3) developing and maintaining an accredited measles laboratory network; and 4) achieving timely identification, investigation, and response to measles outbreaks. This report updates previous reports and summarizes progress toward measles elimination in the South-East Asia Region during 2003–2013 (4). Within the region, coverage with the first dose of a measles-containing vaccine (MCV1) increased from 67% to 78%; an estimated 286 million children (95% of the target population) were vaccinated in SIAs; measles incidence decreased 73%, from 59 to 16 cases per million population; and estimated measles deaths decreased 63%. To achieve measles elimination in the region, additional efforts are needed in countries with <95% 2-dose routine MCV coverage, particularly in India and Indonesia, to strengthen routine immunization services, conduct periodic high-quality SIAs, and strengthen measles case-based surveillance and laboratory diagnosis of measles.

## Immunization Activities

MCV1 was introduced in all 11 countries in the South-East Asia region before 2003. During 2003–2013, MCV1 was administered at age 9 months in all countries except Sri Lanka, where the age of administration was increased from 9 to 12 months in 2011 (Table 1). During 2003–2013, the number of countries in the region with a routine second dose of MCV (MCV2) increased from two to nine. The recommended age for administration for MCV2 varied by country and ranged from 15 months to 7 years. Countries report national and subnational coverage with MCV1 and MCV2 delivered through the routine immunization program to WHO and the United Nations Children’s Fund (UNICEF), which use data from administrative records (vaccine doses administered divided by the target population) and surveys reported by member states each year to estimate MCV1 and MCV2 coverage (5). Estimated MCV1 coverage increased in the region from 67% in 2003 to 78% in 2013; four countries reported ≥95% MCV1 coverage nationwide and in all districts in 2013 (Table 1, Figure). Estimated MCV2 coverage increased from 6% in 2003 to 53% in 2013; in 2013, estimated MCV2 coverage in three countries was ≥95%. During 2003–2013, measles SIAs were conducted in all countries except Thailand and reached 286 million children (95% of target population) (Table 2). Of the 39 SIAs, 16 (41%) achieved ≥95% administrative coverage.

## Surveillance Activities

By 2013, measles surveillance with laboratory confirmation of suspected cases was implemented in all countries in the region. Bangladesh, Nepal, and Myanmar reported case-based measles surveillance data monthly to the WHO South-East Asia Regional Office, whereas other countries in the region reported aggregate measles surveillance data monthly (6). Five countries (Bangladesh, India, Indonesia, Myanmar, and Nepal) used the WHO-supported network of surveillance medical officers initially established for polio eradication to conduct measles surveillance (3). A measles-rubella laboratory network was established in the region by 2003, as an integral part of the WHO Global Measles and Rubella Laboratory Network. By 2013, this regional laboratory network had expanded to include 34 proficient laboratories** with one regional reference laboratory in Thailand. All countries in the region except Timor-Leste had at least one proficient laboratory, including India (nine laboratories), Indonesia (four), and Thailand (13). In addition, Bangladesh, Bhutan, Nepal, Sri Lanka, and Thailand had also started sentinel surveillance for congenital rubella syndrome.

During 2003–2013, a total of 5,680 suspected measles outbreaks were reported in countries in the region, 5,166 (91%) of which were fully investigated.†† Among those investigated, 2,530 (49%) were laboratory-confirmed measles outbreaks, 1,437 (28%) were laboratory-confirmed rubella outbreaks, and 532 (10%) were laboratory-confirmed mixed measles and rubella outbreaks.

## Measles Incidence and Measles Virus Genotypes

From 2003 to 2013, annual measles incidence in the region decreased 73%, from 59 to 16 cases per million population. Five countries reported measles incidence of <5 cases per million in 2013, including three (Bhutan, North Korea, and Maldives) that reported zero cases (Table 1, Figure). In 2013, a total of 248 laboratory-confirmed measles outbreaks and 14 laboratory-confirmed mixed measles and rubella outbreaks were reported in the region. A total of 10,108 confirmed measles cases (laboratory-confirmed and epidemiologically linked) were reported in these outbreaks. The largest proportion of cases (35%) occurred in children aged 1–4 years, followed by children aged 5–9 years (30%), children aged <1 year and persons aged ≥15 years (13% each), and children aged 10–14 years (9%). Of these cases, 68% were in unvaccinated persons. The highest percentage of unvaccinated persons (87%) was in the <1 year age group, followed by the ≥15 years (82%), 10–14 years (71%), 5–9 years (62%) and 1–4 years (61%) age groups.

During 2003–2013, among isolates from patients, measles virus genotypes detected and reported in the region included D4, D7, and D8 in India; D8, D9, G2, and G3 in Indonesia; D5 in Maldives: D5 and D9 in Myanmar; D4 and D8 in Nepal; D8 in Sri Lanka; and D5, D8, D9, and G2 in Thailand.

## Discussion

During 2003–2013, substantial progress was made toward measles control in the South-East Asia Region: through implementation of the regional measles mortality reduction strategies, measles incidence decreased 73% and estimated measles deaths decreased 63% (1,3,7). By 2008, the goal of reducing measles-related deaths by 90% by 2010 from the 2000 baseline was achieved by all countries in the region except India (8,9). After increases in MCV1 and MCV2 coverage and implementation of SIAs, Bhutan, North Korea, and Maldives reported no laboratory-confirmed measles cases in 2013 and might have interrupted endemic measles virus transmission. This apparent success will only be confirmed once the regional verification commission is established and a formal evaluation is conducted, but it indicates that measles elimination in this region is feasible when the current tools and strategies are optimally implemented.

In September 2013, after an extensive review of the progress made and the biologic, programmatic, and financial feasibility of measles and rubella elimination, the 66th session of the Regional Committee of the South-East Asia Region adopted the goal of measles elimination and rubella/congenital rubella syndrome control in the South-East Asia Region by 2020 (1,3), resulting in all six WHO regions now having a measles elimination goal. However, challenges exist to achieving measles elimination in the South-East Asia Region. In 2013, routine MCV1 coverage was <95% nationally for seven of the 11 countries in the region. Of the estimated 21.5 million infants worldwide who did not receive MCV1, almost one third were in India (6.4 million) and Indonesia (0.7 million) (7). In addition, more than half of the SIAs implemented in the region during 2003–2013 did not achieve the target of ≥95% coverage. Information on measles genotypes circulating before measles elimination activities started is important to distinguish indigenous circulating viruses from imported ones, which is required to confirm measles elimination in the region.

The findings in this report are subject to at least two limitations. First, vaccination coverage estimates are derived from administrative data and can be inaccurate because of errors in estimates of target populations or errors in recording doses administered. Second, surveillance data might significantly underestimate actual disease incidence, because not all patients seek care, and not all those who seek care are reported. However, the data on coverage, incidence, and estimated deaths all indicate that measles-associated morbidity and mortality declined considerably in this region during 2003–2013.

What is already known on this topic?

During 1999–2002, coverage with the first dose of measles-containing vaccine in the World Health Organization’s South-East Asia Region increased from 58% to 70%, Sri Lanka and Thailand added a second routine dose of measles-containing vaccine, and 16 million children were vaccinated against measles during supplementary immunization activities (SIAs).

What is added by this report?

During 2003–2013, estimated coverage with the first and second doses of measles-containing vaccine increased from 67% to 78% and from 6% to 53%, respectively, and measles SIAs reached 286 million children. Measles incidence declined by 73%, and estimated measles deaths decreased by 63%. The region adopted the goals of measles elimination and rubella and congenital rubella syndrome control by 2020. All countries in the region conduct some form of case-based measles surveillance, and some countries have implemented sentinel surveillance for congenital rubella syndrome.

What are the implications for public health practice?

To achieve regional measles elimination by 2020, the following are needed: strengthening routine immunization to achieve ≥95% coverage with 2 doses of measles-containing vaccine; optimizing the timing of measles vaccine doses; conducting high-quality SIAs; enhancing surveillance and building on existing laboratory networks; and seeking opportunities for collaboration with other programs.

The adoption of a measles elimination goal in the South-East Asia Region is an opportunity to reenergize efforts and maintain momentum in the region to 1) strengthen routine immunization and achieve ≥95% coverage with MCV2; 2) optimize the timing of MCV1 and MCV2 doses, based on measles epidemiology in each country§§; 3) conduct high-quality SIAs; 4) enhance surveillance and build on existing laboratory networks to perform case-based surveillance; and 5) seek opportunities to collaborate with other programs, including use of the measles elimination platform to integrate rubella and congenital rubella syndrome control efforts. As of 2015, all 11 countries in the South-East Asia Region had either developed or were drafting national plans based on the strategies outlined in the Global Measles and Rubella Strategic Plan and the Regional Committee resolution (1,10). With 35 million surviving infants in the region (26% of the global total), the measles elimination goal is a significant opportunity to further decrease measles-related deaths and illness globally by 2020 (7).

## Figures and Tables

**FIGURE:**
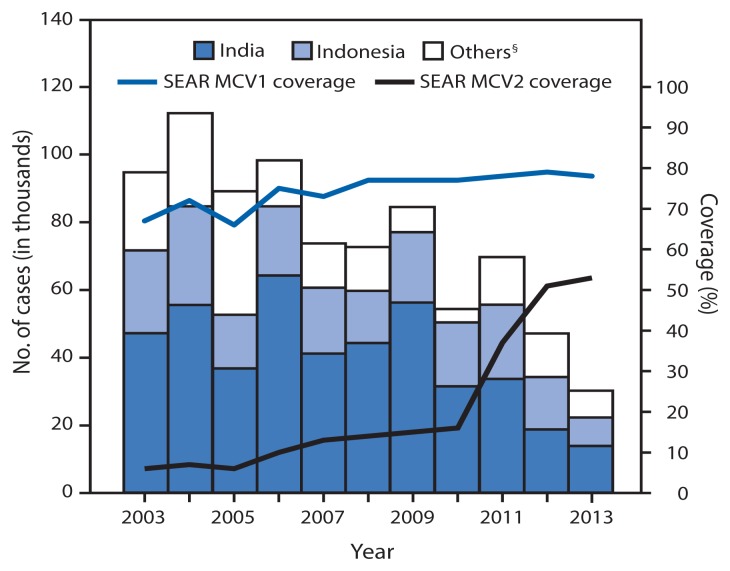
Number of reported measles cases^*^ and estimated percentage of children who received their first and second dose of measles-containing vaccine (MCV),^†^ by country — World Health Organization (WHO) South-East Asia Region (SEAR), 2003–2013 **Abbreviations :** MCV1 = first dose of MCV in routine immunization; MCV2 = second dose of MCV in routine immunization. ^*^ Cases of measles reported to WHO and the United Nations Children’s Fund (UNICEF) through the Joint Reporting Form from WHO-SEAR. ^†^ Data are from WHO and UNICEF estimates for SEAR, available at http://www.who.int/immunization/monitoring_surveillance/data/subject/en. ^§^ Others include Bangladesh, Bhutan, North Korea, Maldives, Myanmar, Nepal, Sri Lanka, Thailand, and Timor-Leste.

**TABLE 1:** Estimated coverage* with the first and second dose of measles-containing vaccine (MCV), vaccination schedule,† number of reported measles cases,§ and measles cases per 1 million population,¶ by country — World Health Organization (WHO) South-East Asia Region, 2003 and 2013

Country	2003						2013						2003–2013	
		
	WHO/UNICEF estimated coverage* (%)		MCV schedule†		No. of reported measles cases (JRF)§	Measles incidence per million population¶	WHO/UNICEF estimated coverage* (%)		MCV schedule†		No. of reported measles cases (JRF)§	Measles incidence per million population¶	% change in MCV1 coverage	% change in measles incidence per million population
			
	MCV1 (%)	MCV2 (%)	MCV1	MCV2			MCV1 (%)	MCV2 (%)	MCV-1	MCV2				
Bangladesh	76	—**	M-9 mos	—**	4,067	30.6	93	81	MR-9 mos	M-15 mos	237	1.5	22	−95
Bhutan	88	—**	M-9 mos	—**	0	00.0	94	89	MR-9 mos	M-24 mos	0	0.0	7	0
North Korea	95	—**	M-9 mos	—**	0	00.0	99	99	M-9 mos	M-15 mos	0	0.0	4	0
India	62	—**	M-9 mos	—**	47,147	44.0	74	42	M-9 mos	M-16–24 mos	13,822	11.1	19	−75
Indonesia	74	21††	M-9 mos	M-7 yrs††	24,457	114.4	84	79	M-9 mos	M-6yrs§§	8,419	33.9	14	−70
Maldives	96	—**	M-9 mos	—**	75	267.3	99	99	M-9 mos	MMR-18 mos	0	—	3	−100
Myanmar	80	—**	M-9 mos	—**	830	15.6	86	80	M-9 mos	M-18 mos	1,010	16.2	8	4
Nepal	75	—**	M-9 mos	—**	13,344	537.8	88	—**	MR-9 mos	—**	1,861	68.3	17	−87
Sri Lanka	99	90	M-9–12 mos¶¶	MR-3 yrs	65	3.4	99	99	MMR-1 yrs	MMR-3 yrs	2,107	102.9	0	2,947
Thailand	96	92	M-9 mos	MMR-6 yrs	4,519	71.8	99	94	MMR-9 mos	MMR-7 yrs	2,641	40.7	3	−43
Timor-Leste	55	—**	M-9 mos	—**	94	110.6	70	—**	M-9 mos	—**	4	3.4	27	−97
**Region overall**	**67**	**6**			**94,598**	**58.9**	**78**	**53**			**30,101**	**16.2**	**16**	−**72**

**Abbreviations:** M = measles; MR = measles-rubella; MMR = measles-mumps-rubella; UNICEF = United Nations Children’s Fund; JRF = Joint Reporting Form.

* Data were from WHO and UNICEF estimates, 2013 revision (as of July 2014). Data available at http://www.who.int/immunization/monitoring_surveillance/data/en.

† As reported to WHO/UNICEF on JRFs for the year.

§ JRF was submitted to WHO and UNICEF by member states with the official immunization data and reports the number of measles cases in the country for the year.

¶ Measles incidence was calculated based on the reported measles cases and population by member states through WHO/UNICEF JRF.

** MCV2 was not introduced in routine immunization.

†† Subnational introduction in schools of West Java at age 7 years.

§§ In a few selected provinces in Indonesia, MCV2 was given at age 24 months.

¶¶ Changed in 2011 from age 9 months to 9–12 months.

**TABLE 2:** Measles supplementary immunization activities (SIAs),* by country, target age group, type of SIA, and number and percentage of targeted children vaccinated — World Health Organization (WHO) South-East Asia Region, 2003–2013†

Country	Year	Vaccine type	SIA type	Extent of SIA	Target age group	Target population	Administrative coverage (%)
Bangladesh	2005	M	Catch-up	Pilot	9 mos–10 yrs	1,481,321	93
	2006	M	Catch-up	National	9 mos–10 yrs	34,199,590	>100§
	2010	M	Follow-up	National	9 mos–5 yrs	18,136,066	100
Bhutan	2006	MR	Catch-up	National	9 mos–14 yrs (males) and 15–44 yrs (females)	338,040	98
North Korea	2007	M	Catch-up	National	6 mos–45 yrs	16,123,376	100
India	2010	M	Catch-up	Subnational	9 mos–10 yrs	10,469,901	90
	2011	M	Catch-up	Subnational	9 mos–10 yrs	34,127,013	90
	2012	M	Catch-up	Subnational	9 mos–10 yrs	50,134,186	90
	2013	M	Catch-up	Subnational	9 mos–10 yrs	36,012,805	93
Indonesia	2003	M	Catch-up	Subnational	6–12 yrs	1,030,445	95
	2004	M	Catch-up	Subnational	6–12 yrs	2,180,918	94
	2005	M	Catch-up	Subnational	6 mos–15 yrs	5,515,324	94
	2006	M	Catch-up	Subnational	6–12 yrs	3,161,323	96
	2006	M	Catch-up	Subnational	6 mos–5 yrs	3,978,096	93
	2007	M	Follow-up	Subnational	6 mos–5 yrs	14,913,092	91
	2007	M	Catch-up	Subnational	6 mos–12 yrs	5,473,025	>100§
	2008	M	Follow-up	Subnational	1–3 yrs	11,203	78
	2009	M	Follow-up	Subnational	9 mos–5 yrs	2,124,572	92
	2010	M	Follow-up	Subnational	9 mos–5 yrs	3,619,024	91
	2011	M	Follow-up	Subnational	9 mos–5 yrs	11,989,559	95
Maldives	2005	MR	Catch-up	National	6–25 yrs (males) and 6–35 yrs (females)	144,997	85
	2006	MR	Catch-up	National	6–25 yrs (males) and 6–35 yrs (females)	144,997	85
	2007	MMR	Catch-up	National	4–6 yrs	29,529	56
Myanmar	2003	M	Follow-up	National	9 mos–5 yrs	2,502,969	90
	2004	M	Follow-up	National	9 mos–5 yrs	1,679,487	65
	2007	M	Follow-up	National	9 mos–5 yrs	6,056,000	94
	2012	M	Follow-up	National	9 mos–5 yrs	6,432,064	97
Nepal	2004	M	Catch-up	National	9 mos–15 yrs	5,344,765	>100§
	2005	M	Catch-up	National	9 mos–15 yrs	4,326,348	>100§
	2008	M	Follow-up	National	9 mos–59 mos	199,751	97
	2008	M	Follow-up	National	9 mos–59 mos	3,903,515	93
	2012	MR	Catch-up	National	9 mos–14 yrs	9,579,306	>100§
Sri Lanka	2003	M	Catch-up	National	10–15 yrs	1,987,847	95
	2004	MR	Catch-up	National	16–20 yrs	1,890,326	72
	2013	M	Catch-up	National	6 mos–12m	176,587	98
Timor-Leste	2003	M	Catch-up	National	9 mos–5 yrs	128,318	99
	2006	M	Catch-up	National	6 mos–14 yrs	390,687	40
	2009	M	Follow-up	National	9 mos–5 yrs	167,136	76
	2011	M	Catch-up	National	6 mos–14 yrs	494,427	92
**Total**						**300,597,935**	

**Abbreviations:** MCV = measles-containing vaccine; M = measles; MR = measles-rubella; MMR = measles-mumps-rubella.

* SIAs generally are carried out using two target age ranges. An initial, nationwide catch-up SIA targets all children aged 9 months–14 years, with the goal of eliminating susceptibility to measles in the general population. Periodic follow-up SIAs then target all children born since the last SIA. Follow-up SIAs generally are conducted nationwide every 2–4 years and target children aged 9–59 months; their goal is to eliminate any measles susceptibility that has developed in recent birth cohorts and to protect children who did not respond to the first measles vaccination.

† Data available at http://www.who.int/immunization/monitoring_surveillance/data/en.

§ Values >100% indicate that the intervention reached more persons than the estimated target population.

## Footnotes

* The WHO South-East Asia Region consists of 11 countries: Bangladesh, Bhutan, North Korea, India, Indonesia, Maldives, Myanmar, Nepal, Sri Lanka, Thailand, and Timor-Leste.

† Measles elimination is defined as the absence of endemic measles cases for a period of ≥12 months in the presence of adequate surveillance. One indicator of measles elimination is a sustained measles incidence of <1 case per 1 million population. Rubella/congenital rubella syndrome control is defined as 95% reduction in disease incidence from the 2013 level.

§ SIAs generally are carried out using two target age ranges. An initial, nationwide catch-up SIA targets all children aged 9 months–14 years, with the goal of eliminating susceptibility to measles in the general population. Periodic follow-up SIAs then target all children born since the last SIA. Follow-up SIAs generally are conducted nationwide every 2–4 years and target children aged 9–59 months; their goal is to eliminate any measles susceptibility that has developed in recent birth cohorts and to protect children who did not respond to the first measles vaccination.

¶ These indicators include 1) ≥2 discarded nonmeasles nonrubella cases per 100,000 population at the national level per year (such cases are defined as suspected cases that have been investigated and discarded as nonmeasles and nonrubella cases using laboratory testing in a proficient laboratory or epidemiologic linkage to a laboratory-confirmed outbreak of another communicable disease that is neither measles nor rubella); 2) ≥2 discarded nonmeasles nonrubella cases per 100,000 per year in ≥80% of subnational administrative units; 3) ≥80% of suspected measles cases tested for measles immunoglobulin M antibodies; 4) ≥80% of suspected cases have an adequate investigation conducted within 48 hours of notification; 5) ≥80% of laboratory-confirmed chains of transmission have adequate samples collected for detecting measles or rubella virus and tested in an accredited laboratory; and 6) an annualized incidence rate of zero for confirmed endemic measles cases.

** A laboratory that has met defined criteria outlined in the report, “Framework for verifying elimination of measles and rubella” (Wkly Epidemiol Rec 2013;88:89–100, available at http://www.who.int/wer/2013/wer8809/en).

†† A house-to-house survey is conducted in the affected area; ≥5 suspected cases serologically tested for measles/rubella immunoglobulin M; and a case investigation form or line list with basic epidemiologic data is completed.

§§ WHO recommendations on optimal age for administration of first and second MCV doses and interval between doses depends on rate of measles transmission in country and capacity of the health systems. Additional information available at http://www.who.int/wer/2009/wer8435.pdf?ua=1.
